# Indications for intraoperative anterior segment optical coherence tomography in corneal surgery

**DOI:** 10.1007/s10792-020-01442-0

**Published:** 2020-06-06

**Authors:** Stefan J. Lang, Sonja Heinzelmann, Daniel Böhringer, Thomas Reinhard, Philip Maier

**Affiliations:** Eye Center, Albert-Ludwigs-University of Freiburg, Killianstr. 5, 79106 Freiburg, Germany

**Keywords:** Anterior segment optical coherence tomography, iOCT, Cornea, DALK, DMEK, Penetrating keratoplasty

## Abstract

**Purpose:**

Recently, intraoperative optical coherence tomography (iOCT) has evolved in the field of ophthalmic surgery. So far, the use of iOCT was mainly focused to lamellar keratoplasty, especially deep anterior lamellar keratoplasty (DALK) and Descemet membrane endothelial keratoplasty (DMEK). The aim of this study was to report our experiences with iOCT to introduce new possibilities of this application.

**Methods:**

We used iOCT in 18 patients who underwent the following surgeries: DALK, DMEK, penetrating keratoplasty, autologous limbal transplantation, transscleral suture fixation of a posterior chamber lens, pannus removal on corneal surface and newborn investigation in Peters’ anomaly. We obtained qualitative video data for all procedures.

**Results:**

With the iOCT, the cannula placement during DALK preparation of the recipient cornea and bubble formation could be visualized to improve the success rate of the big bubble injection. In DMEK, the iOCT enables the visualization of Descemet’s membrane removal in the recipient and graft orientation as well as better control of graft attachment. The iOCT enables intraoperative visualization of the graft–host interface during penetrating keratoplasty. During autologous limbal transplantation, transscleral suture fixation of a posterior chamber lens and removal of corneal surface pannus the iOCT is capable of showing the thickness of lamellar preparations to avoid penetrations and to save healthy recipient’s tissue.

**Conclusion:**

The iOCT is a helpful device for intraoperative anterior segment imaging not only for DALK and DMEK. It is also beneficial in penetrating keratoplasty and every other form of lamellar preparation during corneoscleral surgery.

**Electronic supplementary material:**

The online version of this article (10.1007/s10792-020-01442-0) contains supplementary material, which is available to authorized users.

## Background

Recently, intraoperative optical coherence tomography (iOCT) was established in the field of ophthalmic surgery. The first studies reported on the use of handheld iOCT devices [1, 2]. However, a more feasible option seems to be the use of microscope mounted devices [3]. First reports of intraoperative OCT use were mainly focused on vitreoretinal surgery [3–5], including macular hole surgery [2] and retinal detachment [6].In anterior segment surgery, the use of iOCT was reported for lamellar keratoplasty. In deep anterior lamellar keratoplasty (DALK), the iOCT can be used to visualize the placement of the needle for the injection of the big bubble. Also the preparation of the bare Descemet’s membrane and the visualization of interface fluids can be assisted with iOCT [7, 8]. The use of iOCT for big bubble formation in DALK has been reported [9].

In posterior lamellar keratoplasty, the Prospective Intraoperative and Perioperative Ophthalmic ImagiNg with Optical CoherEncE TomogRaphy (PIONEER) study [10] examined the usefulness of iOCT in Descemet stripping automated endothelial keratoplasty (DSAEK). In this study, the iOCT was used to detect interface fluid during surgery, which correlated with interface opacity [10].

In Descemet membrane endothelial keratoplasty (DMEK), the iOCT can be used to enhance the visibility of the graft and can therefore support graft orientation and unfolding [11]. 

Lately, the iOCT also was introduced to the field of glaucoma surgery by enabling visualization of trabecular aspiration or ab interno trabeculotomy; however, adjustments of wavelengths and oblique scanning are necessary for intraoperative use [12].

The aim of this study is to report our experiences with the new iOCT Rescan® technique in anterior segment surgery, including DALK and DMEK and to evaluate new possibilities of this application.

## Materials and methods

We used the iOCT (OPMI Lumera 700 RESCAN 700, Carl Zeiss Meditec AG, Jena, Germany) in 18 patients. The respective surgeries include two DALK, 8 DMEK, two penetrating keratoplasties, one autologous limbal transplantation, one transscleral suture fixation of a posterior chamber lens, two removals of corneal surface pannus, one examination of Peters’ anomaly in a newborn child and pterygium surgery. The iOCT scans can be directly viewed through the eyepiece of the microscope as well as on the observation monitor, simultaneously to the live surgery images. The scan location marker can be positioned with the foot control panel. The A-scan depth is 2.0 mm, and the axial resolution is 5.5 µm. Scan length can be adjusted between 3 and 16 mm. The scan can be rotated 360°. There are different scan modes available, including 1-line, 5-lines and a cross hair. We obtained the anonymized video data for all procedures. Intra- and postoperative video analysis was performed concerning the importance of the visualization during crucial surgical steps during different surgical procedures. Ethics committee statement for the analysis of anonymized was obtained by the ethics committee of the Albert-Ludwigs-University of Freiburg (10015/19).

## Results

### DALK

**Fig. 1:**
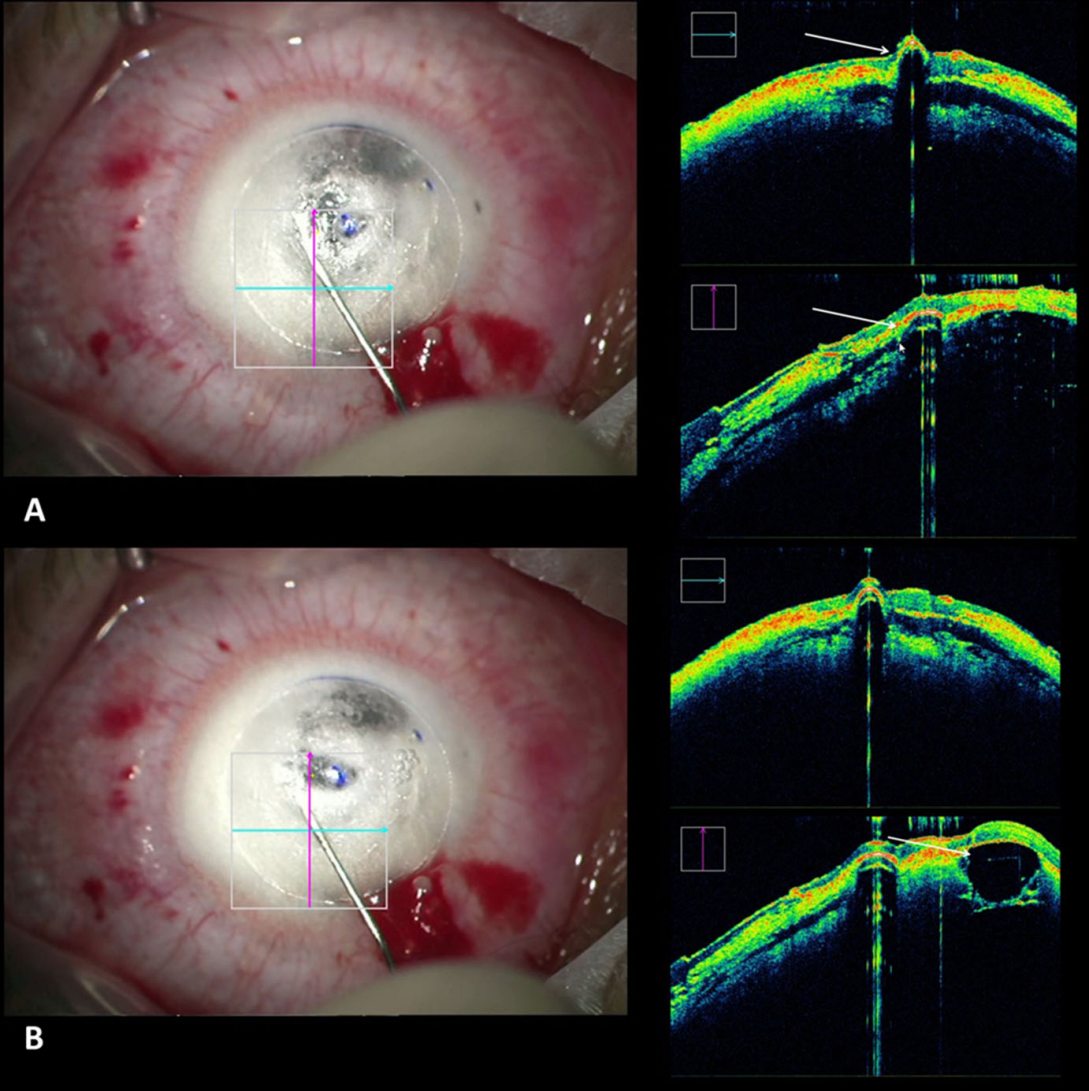
**a** Cannula placement during DALK preparation (arrows). **b** Bubble formation during air injection in DALK (arrow)

With the iOCT, the cannula placement during preparation of the recipient cornea could be visualized although the reflexes of the surgical instruments and the cannula did not always allow for a precise visualization of the cannula’s position in the deep corneal stroma (Fig. 1a). Furthermore, the formation of the big bubble during the air injection was visible (Fig. 1b). Here, the visualization in the peripheral part of the cornea was helpful to see the size of the final big bubble (Video 1). At the end of the surgery, the interface could be evaluated after suturing of the graft (Video 1). Eventual fluid was drained out of the graft–host interface in order to avoid a second anterior chamber in the postsurgical course.

### DMEK

**Fig. 2:**
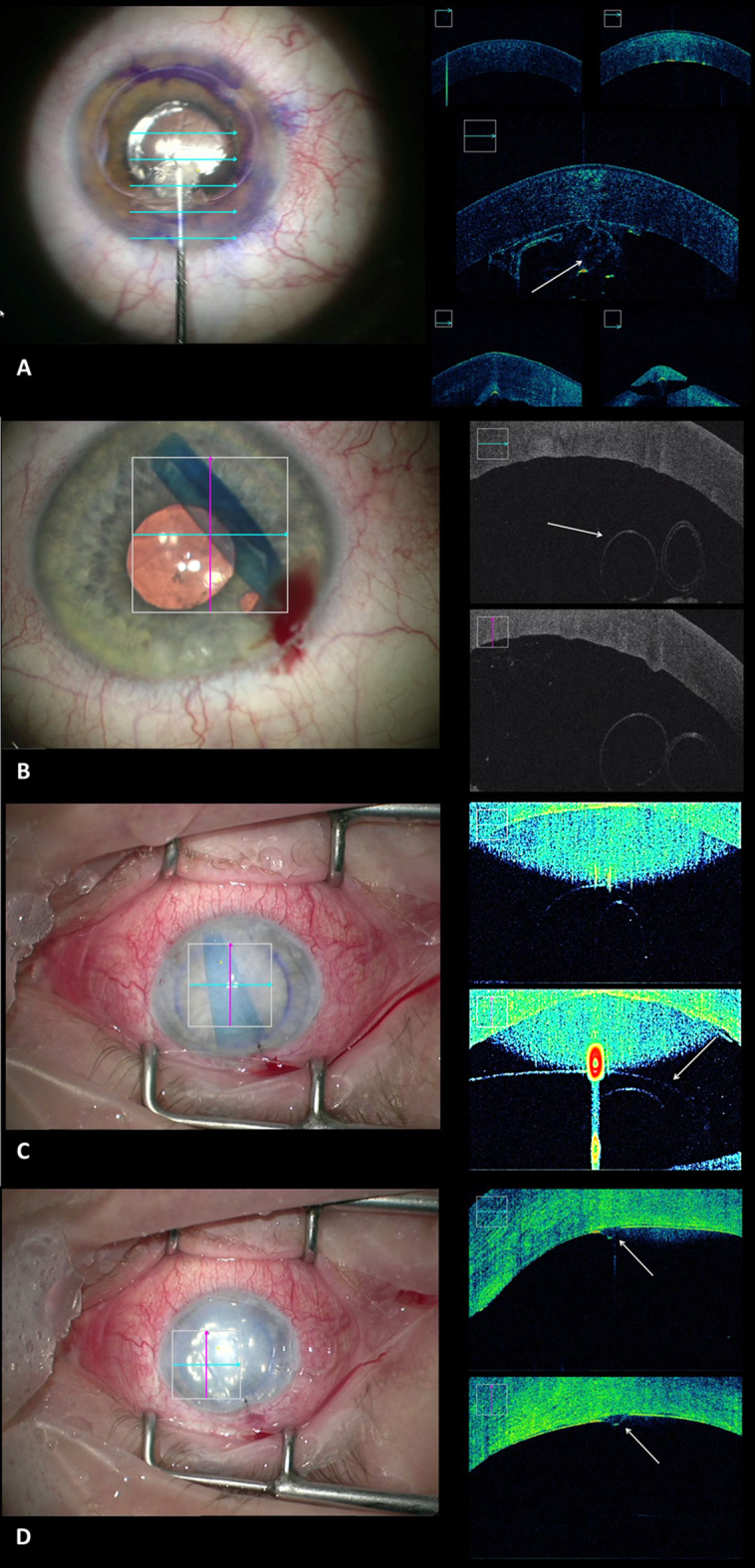
**a** Removal of host Descemet membrane (arrow) for DMEK. **b** Graft orientation in DMEK surgery: The opening of the graft roll is directed to the recipient’s cornea (arrow) indicating the correct orientation of the DMEK graft. **c** Graft orientation in DMEK surgery. The orientation of the graft can clearly be identified even in an opaque cornea. The graft is upside-down as opening of the graft roll is directed to the recipient’s iris (arrow) indicating the incorrect orientation of the DMEK graft. **d** Folds (arrow) in the DMEK graft with low visibility during surgery due to the opaque recipient’s cornea

The iOCT enabled the visualization of the Descemet’s membrane removal in the recipient (Fig. 2a), so that the surgeon could easily control the complete removal in cases of reduced visibility due to corneal opacities. Also, the graft orientation could be evaluated in clear corneas (Fig. 2b) and opaque corneas (Fig. 2c) to avoid upside down attachment of the graft that would lead to primary graft failure. After attaching the graft with an air bubble, the correct position and possible detachments or folds could also be evaluated with the iOCT (Fig. 2d, Video 2).

### Penetrating keratoplasty

**Fig. 3:**
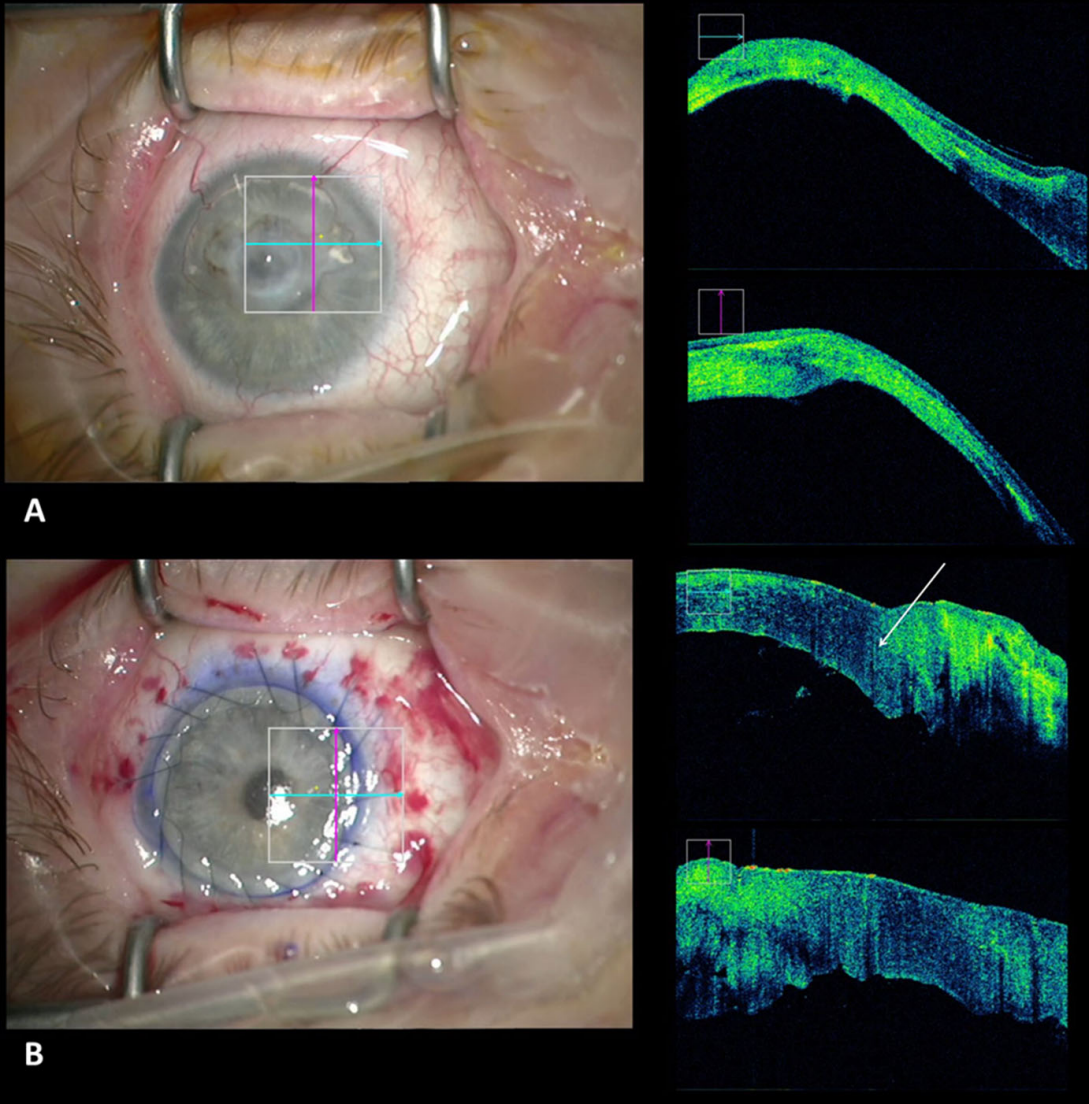
**a** Intraoperative evaluation of the anterior segment in an opaque cornea before penetrating keratoplasty to assess the thickness of the host cornea to determine the best position and size of the trephination to avoid suturing in very thin areas of the recipient’s cornea. **b** Evaluation of the graft–host interface at the end of penetrating keratoplasty (arrow)

During penetrating keratoplasty, the iOCT enabled intraoperative visualization of the anterior chamber (Fig. 3a) in cases of reduced visibility to allow for the detection and interpretation for, e.g., anterior synechiae or thickness of the recipient’s cornea for best positioning of the trephination (Video 3). After suturing of the graft, the iOCT could be used to visualize the graft–host interface (Fig. 3b) to allow for the detection of wound dehiscence (Video 3).

### Autologous limbal transplantation

**Fig. 4:**
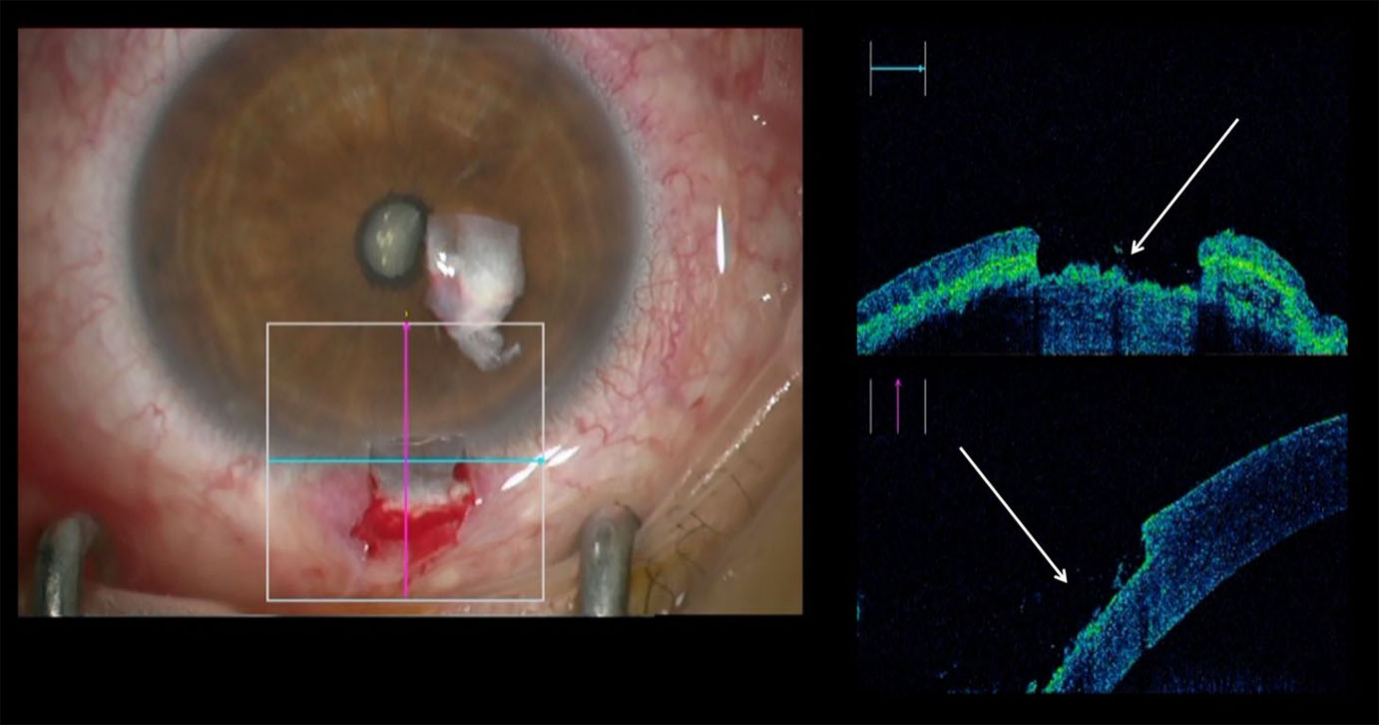
Evaluation of the depth of the donor site in autologous limbal transplantation to assure the transplantation of the limbal niche (arrows)

During graft preparation on the healthy eye of a patient with complete unilateral limbal stem cell deficiency, the depth of the lamellar preparation could be assessed with the iOCT (Fig. 4) to assure that enough limbal tissue with the respective niche could be obtained. During graft suturing on the limbal deficient eye, the depth of the pockets where the grafts are placed could also be visualized to allow for best attachment.

### Transscleral suture fixation of a posterior chamber lens

**Fig. 5:**
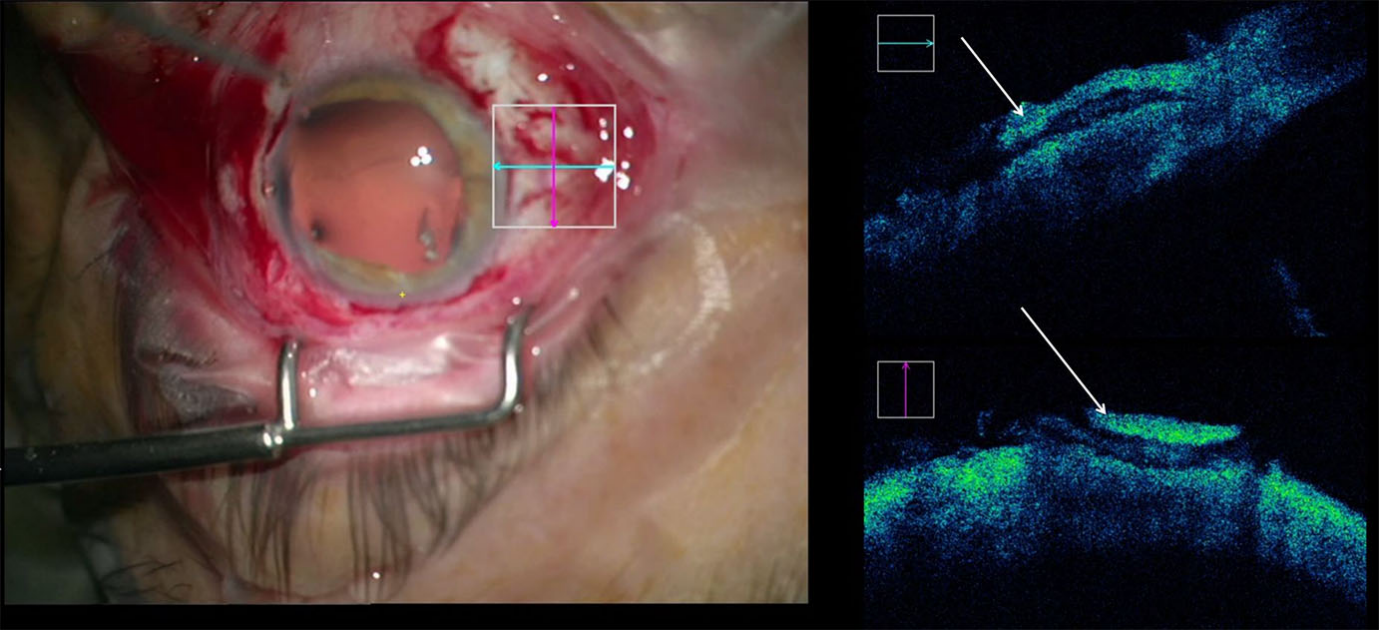
Flap preparation in transscleral suture fixation of a posterior chamber lens (arrows point at the flap)

With the iOCT, the flap preparation of the sclera, for the transscleral sutures underneath, could be visualized, enabling evaluation of the preparation depth (Fig. 5) to allow for save and firm fixation of the IOL.

### Removal of corneal surface pannus

**Fig. 6:**
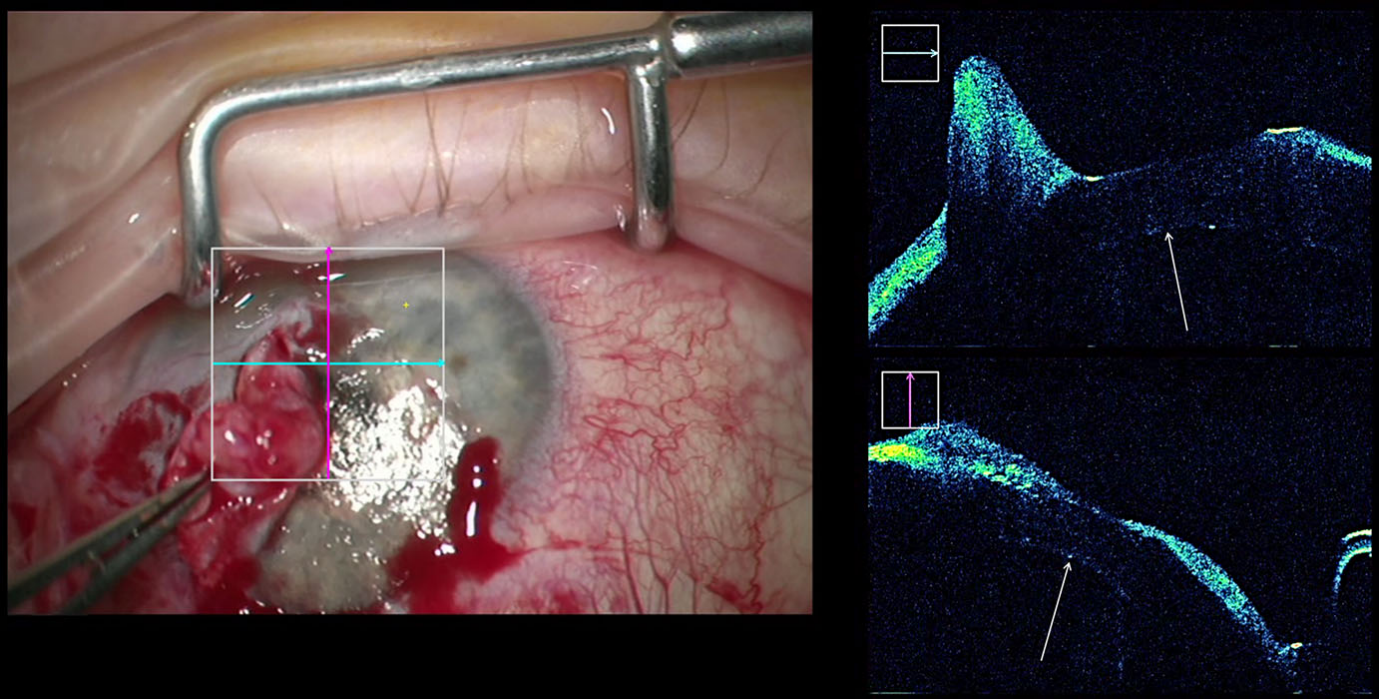
Evaluation of preparation depth during corneal pannus removal to avoid penetration of the globe as the remaining corneal thickness cannot be seen through the surgical microscope (arrows points at the posterior cornea)

Intraoperative scanning of the pannus and the remaining stroma was possible with the iOCT (Fig. 6) which helps to allow for complete removal of the scar tissue and simultaneously to avoid penetrations of the globe as the depth of the preparation can hardly be seen through a surgical microscope (Video 4).

### Peters’ anomaly

**Fig. 7:**
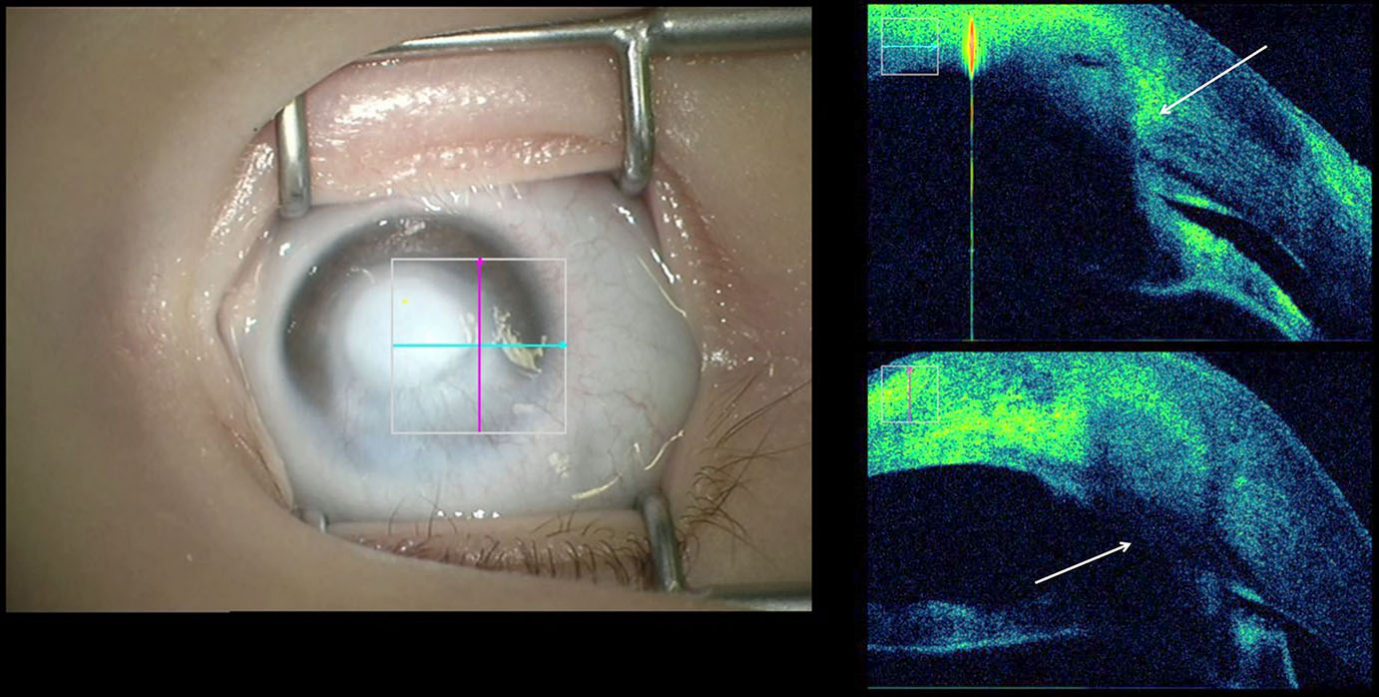
Evaluation of a newborn’s cornea with Peters’ anomaly showing anterior synechia (arrows)

The iOCT enabled the surgeon to evaluate the anterior chamber with possible anterior synechia and attachment between lens and cornea in a newborn even though the cornea was completely opaque (Fig. 7, Video 5). These pieces of information can hardly be obtained in newborn infants with current OCT devices as general anesthesia is needed and are necessary for further decisions regarding surgical interventions like penetrating keratoplasty, lensectomy or glaucoma surgery.

### Excision of a pterygium

**Fig. 8:**
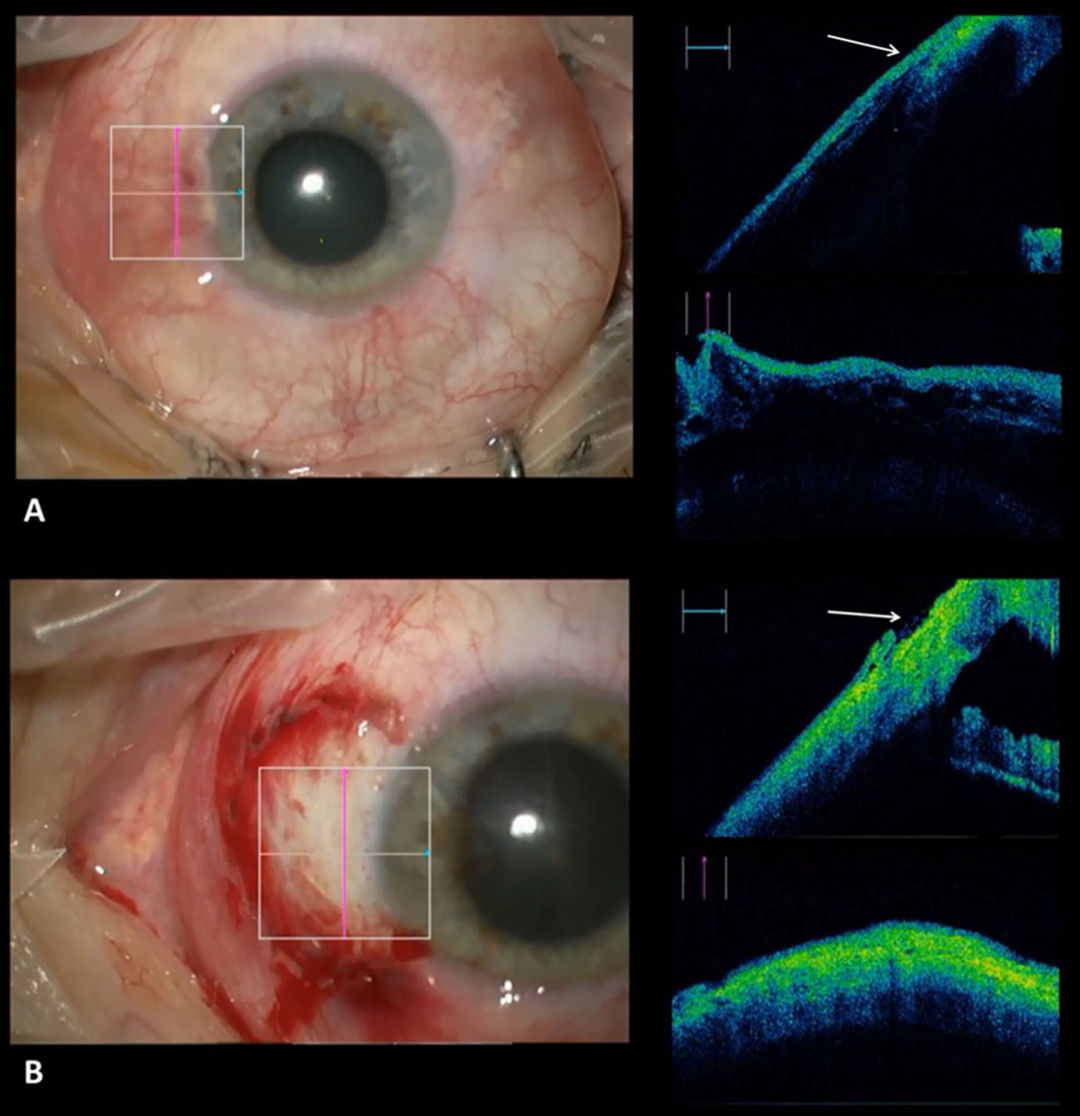
**a** Visualization of the pterygium and cornea before surgical removal (the base of the pterygium at the limbus is marked with an arrow). **b** Bare sclera after removal of the pterygium (arrow)

During surgery, the iOCT could visualize the pterygium with the depth of its attachment to the cornea (Fig. 8a). This can prevent a corneoscleral perforation. The iOCT could also be used to evaluate the field of the bare sclera of the removal site after the excision where the conjunctival graft is placed (Fig. 8b).

## Discussion

The iOCT used in this case series enabled visualization of the complete anterior segment assisting far more surgical procedures than the previous reported DALK, DMEK and DSAEK including sclera for example. The visualization of the cannula and the bubble formation during DALK preparation can indeed be helpful for the surgeon, especially since the cornea may become opaque during the air injection. This is concordant to the findings of previous studies [7, 13]. The big bubble formation was assessed in a retrospective study in 100 eyes by Scorcia et al. [13]. They described that depending on the iOCT findings a repositioning of the cannula may improve the outcome of DALK surgery [13]. The combination with femto-second laser has also been reported in the literature [14]. The technique of deep trephination for DALK enhances the success rate of the type 1 bubble formation [15]. Possibly, the additional depth information gained from iOCT might further increase the success rate. In cases where iOCT is not possible or does not deliver adequate information for cannula placement, the red reflex can help to locate the depth of the cannula when penetrating the stroma as described by Scorcia et al. [16].

The share of posterior lamellar keratoplasty is growing in the recent years [17], and regular anterior segment OCT is already established in postoperative examination of posterior lamellar keratoplasty [18]. During DMEK surgery, the iOCT has been reported to help with the graft orientation and the unfolding of the graft [11]. We found this especially helpful in cases with corneal edema and impaired visibility for the surgeon. Beneficial effects of iOCT have also been described by other authors [19, 20]. Possible beneficial effects regarding the rebubbling rate should be evaluated in studies comparing iOCT to standard methods.

Also, conventional penetrating keratoplasty may benefit from the use of iOCT. The evaluation of the corneal stroma and the anterior chamber can be helpful in cases of scarred and therefore opaque corneas to find the best trephination diameter and position in order to allow for safe suturing of the graft by avoiding trephination in thin areas of the recipient’s cornea. Also, the visualization of the interface during and after suturing may be helpful in special cases.

Furthermore, the iOCT provides the opportunity of live assessment of the preparation depth in all cases of lamellar preparation in the anterior segment. This includes scleral flap preparation that is done for transscleral suture fixation of a posterior chamber lens or the lamellar graft preparation for an autologous limbal tissue graft. Corneal pannus or pterygium can be visualized during preparation to allow for an exact preparation depth to remove the diseased tissue completely and to avoid penetration of the globe. This may be especially helpful in cases of severe recurrent pterygium with deep infiltration of the cornea and the sclera and even in performing corneal biopsies [21]. The iOCT provides immediate visual feedback to the surgeon during the surgery and may prevent accidental perforation, especially in complicated surgical situations. In contrast to conventional OCT devices, the mounted iOCT device also enables anterior segment imaging in lying patients like newborn or infants. This is also helpful for uncooperative patients that can only be examined in general anesthesia.

Disadvantages of the iOCT tested in this case series are the need for an additional person in the operating room, handling the iOCT. This might improve as all surgeons were at the beginning of their personal learning curve using iOCT. Furthermore, the iOCT-operator had to describe the OCT pictures to the surgeon in certain complicated surgical situations. Finally, the additional costs of the system have to be taken into account. 

In conclusion, the iOCT is a helpful device, not only for vitreoretinal surgery [3, 4], but also for anterior segment imaging [7, 11, 22] and child/newborn examination. The range of surgical techniques that may improve does not only include DALK and DMEK, but also conventional penetrating keratoplasty and every kind of lamellar preparation like in autologous limbal transplantation or pannus, pterygium or tumor removal.

## Electronic supplementary material

Below is the link to the electronic supplementary material.

Video 1. DALK: 0:00–0:26: Cannula placement and big bubble formation. 0:26–1:14: Visualization of Descemet’s membrane during excision of the anterior corneal stroma. (MP4 45484 kb)

Video 2. DMEK: 0:00–0:25: Visualization of the graft orientation inside the anterior chamber. 0:25–0:51: Visualization of the graft–host interface during air injection to attach the graft. (MP4 44039 kb)

Video 3. PKP: 0:00–0:30: Evaluation of the graft thickness and anterior chamber to find the best trephination diameter and the best place for trephining the recipient’s cornea. 0:30–0:33: Visualization of the wound area after suturing of the graft. (MP4 24752 kb)

Video 4. Removal of corneal surface pannus: 0:00–0:07: Evaluation of the pannus and the thickness of the residual corneal stroma underneath. 0:07–0:21: Corneal stroma evaluation during pannus removal. 0:21–0:39: Corneal stroma and sclera thickness after pannus removal. (MP4 26980 kb)

Video 5. Visualization of anterior synechiae in the anterior chamber in a patient with Peters’ anomaly through an opaque cornea. (MP4 17030 kb)

## References

[1] Hahn P, Migacz J, O’Connell R (2011). The use of optical coherence tomography in intraoperative ophthalmic imaging. Ophthalmic Surg Lasers Imaging.

[2] Wykoff CC, Berrocal AM, Schefler AC (2010). Intraoperative OCT of a full-thickness macular hole before and after internal limiting membrane peeling. Ophthalmic Surg Lasers Imaging.

[3] Tao YK, Ehlers JP, Toth CA, Izatt JA (2010). Intraoperative spectral domain optical coherence tomography for vitreoretinal surgery. Opt Lett.

[4] Ehlers JP, Tao YK, Srivastava SK (2014). The value of intraoperative optical coherence tomography imaging in vitreoretinal surgery. Curr Opin Ophthalmol.

[5] Inoue M, Itoh Y, Koto T (2019). Intraoperative OCT Findings May Predict Postoperative Visual Outcome in Eyes with Idiopathic Macular Hole. Ophthalmol Retina.

[6] Ehlers JP, Ohr MP, Kaiser PK, Srivastava SK (2013). Novel microarchitectural dynamics in rhegmatogenous retinal detachments identified with intraoperative optical coherence tomography. Retina (Philadelphia, Pa).

[7] Steven P, Le Blanc C, Lankenau E (2014). Optimising deep anterior lamellar keratoplasty (DALK) using intraoperative online optical coherence tomography (iOCT). Br J Ophthalmol.

[8] Siebelmann S, Steven P, Cursiefen C (2016). Intraoperative Optical Coherence Tomography In Deep Anterior Lamellar Keratoplasty. Klin Monbl Augenheilkd.

[9] Myerscough J, Friehmann A, Busin M, Goor D (2019). Successful Visualization of a Big Bubble during Deep Anterior Lamellar Keratoplasty using Intraoperative OCT. Ophthalmology.

[10] Juthani VV, Goshe JM, Srivastava SK, Ehlers JP (2014). Association between transient interface fluid on intraoperative OCT and textural interface opacity after DSAEK surgery in the PIONEER study. Cornea.

[11] Steven P, Le Blanc C, Velten K (2013). Optimizing descemet membrane endothelial keratoplasty using intraoperative optical coherence tomography. JAMA Ophthalmol.

[12] Heindl LM, Siebelmann S, Dietlein T (2014). Future prospects: assessment of intraoperative optical coherence tomography in Ab interno glaucoma surgery. Curr Eye Res.

[13] Scorcia V, Busin M, Lucisano A (2013). Anterior segment optical coherence tomography-guided big-bubble technique. Ophthalmology.

[14] Liu Y-C, Wittwer VV, Yusoff NZM (2019). Intraoperative optical coherence tomography-guided femtosecond laser-assisted deep anterior lamellar keratoplasty. Cornea.

[15] Myerscough J, Bovone C, Scorcia V (2019). Deep trephination allows high rates of successful pneumatic dissection for DALK independent of surgical experience. Cornea.

[16] Scorcia V, Lucisano A, Pietropaolo R (2015). Red reflex-guided big-bubble deep anterior lamellar keratoplasty: a simple technique to judge dissection depth. Cornea.

[17] Lang SJ, Bischoff M, Böhringer D (2014). Analysis of the changes in keratoplasty indications and preferred techniques. PLoS ONE.

[18] Heinzelmann S, Böhringer D, Maier PC, Reinhard T (2014). Correlation between visual acuity and interface reflectivity measured by pentacam following DSAEK. Acta Ophthalmol.

[19] Patel AS, Goshe JM, Srivastava SK, Ehlers JP (2020). Intraoperative optical coherence tomography-assisted descemet membrane endothelial keratoplasty in the discover study: first 100 cases. Am J Ophthalmol.

[20] Muijzer MB, Soeters N, Godefrooij DA (2020). Intraoperative optical coherence tomography-assisted descemet membrane endothelial keratoplasty: toward more efficient safer surgery. Cornea.

[21] Schmidt EM, Stiefel HC, Houghton DC, Chamberlain WD (2019). Intraoperative Optical Coherence Tomography to Guide Corneal Biopsy: A Case Report. Cornea.

[22] Augustin AJ (2018). Intraoperative optical coherence tomography - an overview of current clinical data for the application in the anterior and posterior segments. Klin Monbl Augenheilkd.

